# Content validity of SarQoL, a quality of life questionnaire specific to sarcopenia

**DOI:** 10.1007/s40520-024-02756-0

**Published:** 2024-04-30

**Authors:** C. Demonceau, B. Voz, O. Bruyère, J-Y. Reginster, C. Beaudart

**Affiliations:** 1 Department of Public Health, University of Liège, Liege, Belgium; 2 College of Science, King Saud University, Riyadh, Saudi Arabia; 3 Clinical Pharmacology and Toxicology Research Unit, Department of Biomedical Sciences, Faculty of Medicine, NAmur Research Institute for LIfe Sciences (NARILIS), University of Namur, Namur, Belgium

**Keywords:** Sarcopenia, Health-related quality of life, SarQoL, Content validity

## Abstract

**Background:**

The Sarcopenia & Quality of Life (SarQoL) questionnaire is a patient-reported outcome measure designed for assessing health-related quality of life in individuals with sarcopenia. Despite its wide acceptance in the scientific literature, its content validity has only been partially demonstrated so far.

**Aims:**

To enhance the evidence supporting the content validity of the SarQoL questionnaire.

**Methods:**

Following COSMIN methodology, semi-structured interviews were conducted with 17 Belgian older adults who met the EWGSOP2 criteria for the diagnosis of sarcopenia and 11 experts in sarcopenia, with clinical or research background. Comprehensiveness, relevance and comprehensibility of SarQoL content were assessed through individual transcripts and were qualitatively analyzed thematically according to the seven dimensions of SarQoL.

**Results:**

The majority of the concepts elicited during the semi-structured interviews fitted within existing SarQoL dimensions. Importantly, the different domains of SarQoL were consensually considered as relevant by patients and experts. Some new emergent concepts were identified by the participants. While many of them could be considered as enrichments of existing dimensions or sub-concepts, other new concepts (i.e. self-fulfilment, acceptance of the reduced condition, adaptation/use of strategies, depression) may highlight two potential dimensions not covered by SarQoL, i.e. patient empowerment and depression. Cognitive interviews also highlighted that SarQoL items and instructions were clear and comprehensible.

**Conclusions:**

SarQoL, in its current form, demonstrates good evidence of content validity for assessing health-related quality of life in patients with sarcopenia. We do not recommend adding new items or dimensions to SarQoL. Instead, for researchers or clinicians who aim to specifically address self-empowerment or depression of sarcopenic populations, we suggest completing the assessment of quality of life by concurrently using additional validated scales of patient empowerment or depression.

**Supplementary Information:**

The online version contains supplementary material available at 10.1007/s40520-024-02756-0.

## Introduction

Sarcopenia, defined as a progressive decline in muscle mass and muscle strength, the severity of which is determined by physical performance [1], has been widely highlighted in the scientific literature for its impact on health-related quality of life (HRQoL) [2]. In 2013, a HRQoL sarcopenia-specific patient-reported outcome measure (PROM), namely the Sarcopenia & Quality of Life (SarQoL) questionnaire, has been developed. Indeed, while generic tools (e.g. SF-36, EQ-5D) were previously used to measure HRQoL in sarcopenic individuals, specific instruments have been shown to be associated with greater validity, credibility and responsiveness to change at the individual level and are widespread due to their ability to capture patient experiences that cannot be measured by traditional physiological outcomes [3]. Today, the SarQoL questionnaire, available in more than 30 languages (www.sarqol.org) is the unique available HRQoL specific questionnaire for sarcopenia. Nineteen validation studies performed on SarQoL have consensually confirmed the capacity of SarQoL to detect difference in HRQoL between older people with and without sarcopenia, its reliability and its validity [4].

Although SarQoL is widely accepted and used, one of its psychometric properties, i.e. content validity, is considered as insufficient according to the COSMIN guidelines published in 2018 [5]. The reason is that, since the initial development of the SarQoL questionnaire in 2013, more detailed guidelines and standards have been established for demonstrating PROMs’ content validity. Demonstrating the content validity of a PROM requires to generate qualitative evidence that ensures that the questionnaire’s framework and items align effectively with the intended measurement concept, population and context of use [6]. The development of SarQoL was based on an exhaustive literature review, experts and sarcopenic patients’ interviews, from which a list of 180 potential items was generated. Professionals and sarcopenic patients were then asked to review the list and select the items they considered most relevant to be included in SarQoL. After that, a pre-test of SarQoL was conducted with 21 sarcopenic individuals that confirmed the comprehensibility and comprehensiveness of the tool [5, 7]. Nevertheless, concerning the content validity itself, COSMIN methodology requires to evaluated criteria encompassing 3 major aspects: relevance, comprehensibility and comprehensiveness in patients and experts [5]. To date, only one study has conducted a content validity analysis per se of SarQoL [8]. While the results of this study highlighted an adequate and acceptable content validity, this study was based solely on professionals’ opinions without fulfilling all the COSMIN criteria and without any patient involvement, leaving the content validity of SarQoL not entirely confirmed according to the COSMIN criteria [7]. .

The current study aimed to fill this gap to provide a complete assessment of content validity of SarQoL by conducting qualitative interviews with experts and patients suffering from sarcopenia.

## Methods

This study has been approved by the Ethics Committee of the Teaching Hospital of the University of Liège in June 2023 with an amendment to a previous study protocol (reference 2012/277). Prior to the interviews, all patients provided written informed consent. The specific protocol related to this research is available on Open Science Framework (https://osf.io/6swue/). The COSMIN standard for validating the content validity of a PROM was followed thorough the whole conduct of this research [5].

### The SarQoL questionnaire

SarQoL consists of 55 items integrated into 22 questions covering 7 dimensions of HRQoL: physical and mental health (Domain 1 (D1), locomotion (D2), body composition (D3), functionality (D4), activities of daily living (D5), leisure activities (D6) and fears (D7). Most of these items can be thought of as 3-, 4-, or 5-point Likert items, whereas the remaining are multiple choice questions that permit more than one answers. The questionnaire gives a score for each of these dimensions as well as an overall score out of 100 points. Higher scores reflect better HRQoL.

### Participants

#### Patients

Patients were recruited within the SarcoPhAge (Sarcopenia and Physical Impairment with Advancing Age) cohort [9]. This cohort consists of French-speaking Belgian community-dwelling older adults (65 years and older), followed annually since 2013.

For the assessment of content validity of SarQoL, patients diagnosed with sarcopenia, according to EWGSOP2 criteria, were contacted to be part of the research [1]. Specifically, the sarcopenic patients in this cohort were invited to participate in this study on a step-by-step basis until the data saturation was reached. Efforts were made to recruit participants representing the full spectrum of sarcopenic patients, including those suffering from severe sarcopenia. In line with the COSMIN methodology and based on original studies with similar aims to this study [5, 10, 11], we set an initial sample size of 20 participants as the target sample. However, to ensure adequacy, concept saturation was examined [5] and interviews were conducted until complete concept saturation was observed.

#### Experts

Eleven experts with clinical or research experience in the field of sarcopenia were recruited to evaluate the relevance, comprehensiveness, and comprehensibility of SarQoL. The panel consisted of five clinical researchers, one gerontologist, four geriatricians, one intensive care physician and one cardiologist, ensuring a diversity of expertise. Interviews were conducted either in French or English, depending on the language preference of the expert [5].

#### Qualitative interviews

A semi-structured interview guide (available on the OSF account related to this project https://osf.io/6swue/) was developed based on the ten criteria for good content validity of a PROM recommended by the COSMIN guideline [5] as well as on the interview guide developed by the EORTC Quality of Life Working Group who recently published a study with similar objectives to our study [10]. Two trained researchers team member (CD, CB) conducted face-to-face interviews with participants and experts and recorded them with their consent. All interviews began with an open discussion to allow participants to describe how sarcopenia may affect their HRQoL. This ensures a free and open generation of concepts. Afterward, SarQoL was thoroughly reviewed to explore the relevance of the seven domains to ensure that each domain was explored in detail. The final step of the interview consisted of a structured cognitive debriefing on the comprehensibility and comprehensiveness of each question of SarQoL. Participants were also asked to provide their feedback on their understanding of the instructions and their opinion on the length of the SarQoL questionnaire.

### Data analysis

The content validity of SarQoL was assessed through a qualitative analysis of interview data. Audio recordings were integrally transcribed and anonymised. A thematic framework, based on the seven dimensions originally established in the SarQoL questionnaire, was used as the conceptual framework, using NVivo software. Verbatims of the interviews were categorised into the corresponding framework sections. Any newly identified elements not included in the original SarQoL framework were integrated in the framework if they met to the criteria of homogeneity, objectivity, exclusivity and relevancy [12]. As recommended by the COSMIN methodology, in addition to involving researchers with experience in the domain of sarcopenia and the construct of interest, an additional qualitative researcher (BV) with experience of PROM development, who was not part of the development of the questionnaire, was involved in the analysis to ensure the quality and objectivity of the analysis [5]. To ensure consistency and appropriate interpretation of the thematic framework, a quality control of 10% of the transcriptions was initially analysed independently by two researchers (CD, BV) with subsequent cross-checking to ensure the accuracy of completion. Other transcripts were cross-checked in case of inconsistencies or uncertainties.

Comprehensiveness was determined by eliciting the verbatims of the open discussion to ensure that key concepts or dimensions associated with HRQoL in sarcopenia were covered by SarQoL. A dimension was considered to be elicited if the participants expressed that this dimension impacts HRQoL. Concepts elicited by at least 2 patients or 2 experts were considered in the thematic mapping. The relevance of SarQoL was assessed by going through the questionnaire with participants. For patient’s interviews, an item was considered as relevant when participants expressed either a bit difficulties, difficulties or incapacity of performing this item. For experts, relevance of each individual items was measured on a scale ranging from 1-not relevant to 4-very relevant. The comprehensibility of SarQoL was measured by the comprehensibility of the instructions, the questions and the responses items. For patient’s interviews, comprehensibility was considered as good when patients expressed verbal evidence of their comprehension of each question with a relevant answer. Experts, on the other side, were asked to rate their comprehensibility of each item on a scale ranging from 1-not comprehensible to 4-very clear and comprehensible.

A cognitive debriefing was then performed to investigate the opinion of participants regarding the length of the questionnaire and the order of questions. It was also asked if participants considered that any items should be deleted from the questionnaire, or if any item could be missing to offer a comprehensive assessment of the impact of sarcopenia on HRQoL.

## Results

### Participants characteristics

A total of 28 interviews (i.e., 17 with patients and 11 with experts) were conducted. Interviews lasted from 10 to 62 min. Data saturation was reached during interviews, indicating that no new dimensions or concepts were introduced to complete the framework during the last interviews with patients. Sarcopenic patients had a mean age of 82 ± 6.4 years and 75.4% of the sample were women. They took a mean of 4.8 ± 2.8 medications per day and had 2.9 ± 3.0 concomitant diseases. All patients (100%) were diagnosed with sarcopenia according to EWGSOP2 criteria and 23.5% were also severe sarcopenic. Expert panel was composed with five clinical researchers, one gerontologist, four geriatricians, one intensive care physician and one cardiologist. Experts were from Belgium, Spain, France, Germany and Saudi Arabia, 8 were women, and they had a mean professional experience of 9.6 ± 4.8 years with sarcopenia.

### Comprehensiveness

#### Patients

During the 17 interviews where participants discussed their experience of sarcopenia in daily life and the impact of sarcopenia on their HRQoL, six out of the seven dimensions present in the SarQoL questionnaire were spontaneously elicited by at least 2 patients. The thematic mapping showed that 58.9% of the participants mentioned an impact of sarcopenia on their activities of daily living (Domain 5 (D5) of SarQoL), 41.2% on their leisure activities (D6 of SarQol), 23.5% on their locomotion (D2 of SarQoL), 23.5% on fears (D7 of SarQoL) 17.6% on their physical and mental health (D1 of SarQoL) and 17.6% on their functionality (D4 of). Only the D3, i.e. body composition, was mentioned by one unique patient. In addition, some additional concepts or dimensions emerged during the interviews. While some of them, such as the need for assistance (reported by 23.5% of participants) and the fear of the future (reported by 11.8% of them) were already covered by the existing mapping framework, three other concepts (arbitrarily named), i.e. adaptation and use of strategies (reported by 47.1%), self-fulfillment (reported by 29.4%) and acceptance of the reduced condition (reported by 11.8%) could be considered as distinct from the framework, highlighting one domain not covered by SarQoL, the domain of “patient empowerment”.

#### Experts

Out of the 11 interviews where experts discussed the consequences of sarcopenia on HRQoL based on their clinical or research experience, 6 out of the 7 dimensions of SarQoL were spontaneously elicited by at least two experts. While the dimension of body composition was not elicited (D3 of SarQoL; 0%), other dimensions, namely, activities of daily living (D5 of SarQoL; 100% of experts), physical and mental health (D1 of SarQoL; 73%), leisure activities (D6; of SarQoL 64%), locomotion (D2 of SarQoL; 45%), functionality (D4 of SarQoL; 45%) and fears (D7 of SarQoL; 27%) were elicited according to the thematic mapping. During the assessment of the comprehensiveness of SarQoL, additional concepts or domains emerged. While some of them could once again be considered as already covered by the thematic framework (i.e. hygiene cares and social isolation, reported by 36% of the experts respectively), two new concepts, namely “depression” (reported by 27% of the experts) and “increased dependency” (reported by 36% of them), were not covered by the thematic framework of the SarQoL questionnaire. These concepts could be integrated into new dimensions, depression and patient empowerment.

After reviewing the SarQoL questionnaire, experts were asked if they considered that any item could be missing to assess the quality of life in sarcopenic patients. Six experts (i.e., 54.5%) highlighted depression as the only missing item.

A detailed analysis of items/concepts elicited by at least two patients or experts is available in the supplementary material (Table 1).

### Relevance

#### Patients

**Fig. 1:**
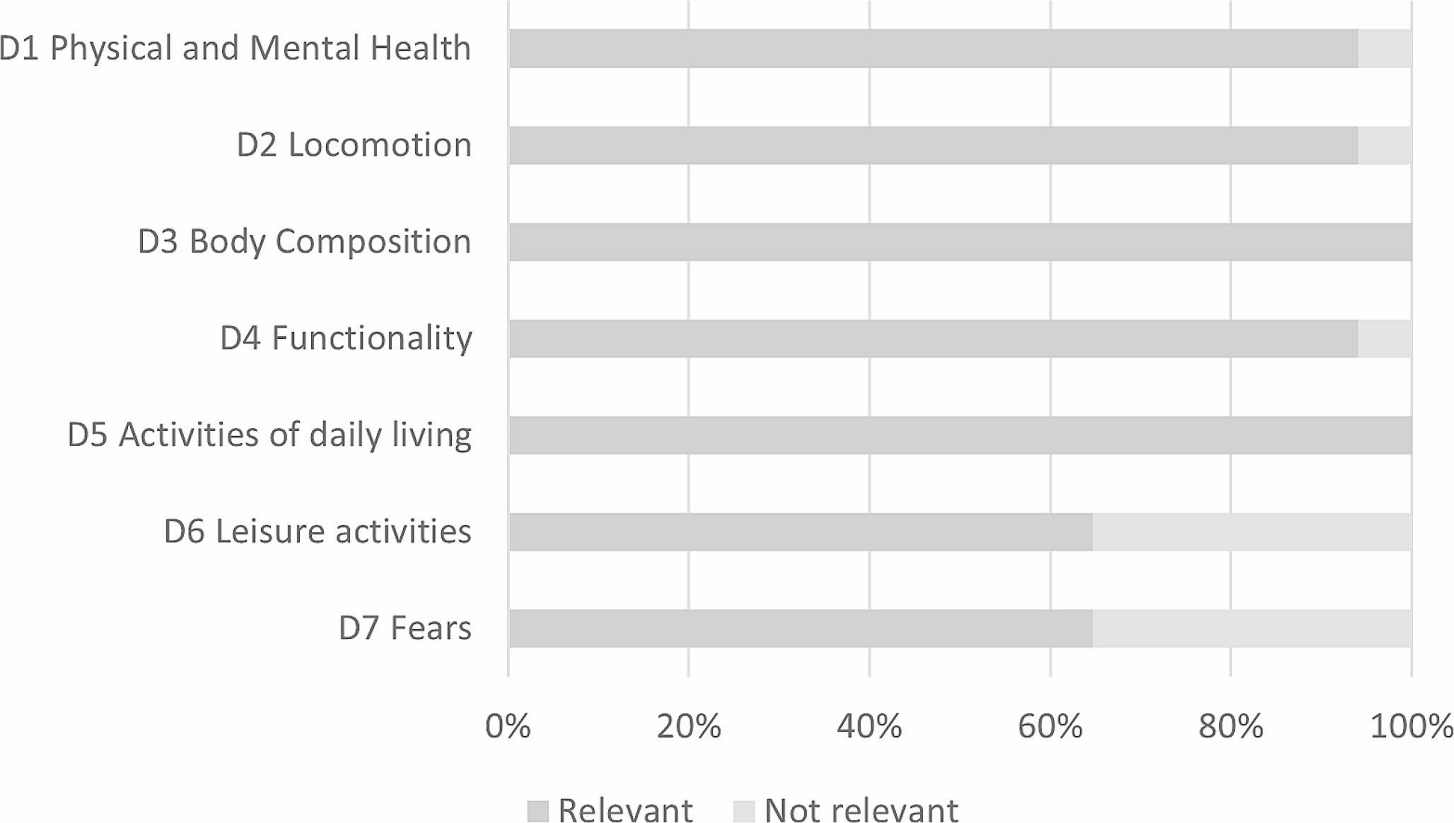
Proportion of patients reporting SarQoL dimensions as relevant to their experience of sarcopenia (*n* = 17)

Patients judged the items of SarQoL, displayed under seven dimensions, as relevant. The relevance of the dimensions ranged from 65% (i.e. fears, leisure activities) to 100% (i.e. activities of daily living, body composition), with a global average of 87 ± 15% (Fig. 1).

#### Experts

A detailed analysis of the relevance rating of each item on the questionnaire by the experts is available in the supplementary material (Table 2). The mean relevance of each dimension ranged from 3.64 for D3 (body composition) to 3.89 for D7 (fears).

### Comprehensibility

#### Patients

All the participants (100%) expressed a clear understanding of the SarQoL questionnaire instructions, providing evidence that instructions are sufficiently clear and appropriate. No participant suggested that the instructions should be rewritten.

Comprehensibility of the different questions was high. Indeed, only two participants (12%) asked for a clarification of one specific term used in the questionnaire (i.e., physical abilities, movement limitations).

All participants reported that it was easy to select responses to the SarQoL questionnaire and all of them indicated that the length of the questionnaire was appropriate and not too long.

#### Experts

The questionnaire was deemed appropriate by experts, with each question receiving a mean rating between 3.6 and 4. A detailed analysis of the comprehensibility rating of each item by the experts is available in the supplementary material (Table 2).

The length of the questionnaire was judged appropriate by all experts, regardless of their field of activity (clinical or research).

## Discussion

The content validity of a PROM is usually considered as the most important psychometric property as it refers to the relevance and understanding of the target population in a specific context [13]. In 2018, new guidance for establishing content validity of PROM have been published by COSMIN [5]. For being considered as sufficient, content validity should include elicitation of concepts by patients and experts via interviews and should also include an assessment of the understanding of the PROM using cognitive debriefing interviews. Although SarQoL has been widely used for 10 years for assessing HRQoL in patients with sarcopenia [4], it is pertinent to evaluate content validity using current recommended methods, ensuring the concepts assessed are important and relevant to patients with sarcopenia today.

The COSMIN guideline required both patient and professional inputs to ensure the content validity of a PROM. Mahmoodi et al. recently performed a content validity analysis with geriatric experts and reported an acceptable and appropriate content validity of SarQoL [8]. However, this study did not fill all the COSMIN criteria for the content validity of a PROM. Indeed, this study, while emphasizing relevance, overlooked the aspects of comprehensiveness and comprehensibility in assessing the content validity of SarQoL, employing a methodology that diverges from COSMIN standards. Therefore, our study should be considered as filling the gap in content validity assessment of SarQoL, by measuring the content validity in a population of patients with sarcopenia and experts, to be fully adherent to the 2018 COSMIN standard for validating the content validity of a PROM.

This qualitative analysis measured the comprehensiveness, relevance and comprehensibility of SarQoL.

The elicitation of all the dimensions covered by the questionnaire during 17 qualitative interviews with sarcopenic participants and 11 qualitative interviews with experts, using the mapping framework, allowed to confirm the comprehensiveness of the questionnaire. Nevertheless, five new concepts (i.e. higher level of dependance, self-fulfillment, the acceptance of diminished condition, the adaptation or the use of strategies and depression) were elicited by the panel during interviews. 27% of the experts specifically mentioned the concept “higher level of dependence” as relevant. It is important to mention that the development of the original version of SarQoL required the conduct of a systematic literature review, qualitative interviews with five patients and semi-structured interviews with experts. From this, emerged a large list of items (i.e., 180 items), among which, the item “higher level of dependence” was present. However, during the item reduction phase, this item was not rated highly enough to be kept in the final version of SarQoL. Moreover, the concept of “adaptation and use of strategies”, as well as the concepts of “personal fulfilment” and “acceptance of the reduced condition”, mentioned by patients, can be encompassed in the dimension of “patient empowerment”. According to the conceptual framework developed by Bravo et al, patient empowerment is composed of a core set of indicators, including self-efficacy, sense of meaning and coherence about the condition and attitudes and self-awareness necessary to influence the health behaviour [14]. Interestingly, the definition of these indicators actually corresponds to the three new concepts elicited during the open discussion [14–16]. Patient empowerment is still an emerging concept in the healthcare literature, with no international consensus on its definition and is generally not included in health-related quality of life questionnaires [17]. The fifth new concept, only elicited by the experts, was the concept of “depression”. Once again, this concept was also present in the initial list of items potentially eligible to be included in SarQoL but not rated highly enough during the item reduction phase to be included in the final version of the questionnaire. A recent meta-analysis conducted by Li et al. has highlighted a high prevalence of depression in sarcopenic patients, whatever the criteria used for its diagnosis [18]. This study also highlighted that a multitude of factors could influence the apparition of depression, revealing the difficulty to analyze this aspect in relation with sarcopenia [18]. Expanding the SarQoL questionnaire to include a single question about depression may not adequately address the complexity of this multifactorial condition, which requires additional space and more specific questions to comprehensively capture its impact. This being taken into account and to achieve the aim of balancing patient burden and maintaining the relevance of the questionnaire for all sarcopenic patients, we do not recommend the introduction of new dimensions or concepts in the SarQoL questionnaire. If deemed relevant, it may be appropriate for researchers or clinicians to use additional validated scales to investigate either the patient empowerment, which can encompass more or less concepts depending on the scale used and the characteristics of the population [19] or the construct of depression, such as the Geriatric Depression scale [20]. It is important here to recall that SarQoL can be used alongside other standalone questionnaires to cover additional concepts important and relevant to specific populations. For example, in 2021, the European Society for Clinical and Economic Aspects of Osteoporosis and Osteoarthritis (ESCEO) published, in their recommendations for the conduct of clinical trials for drugs aiming at the treatment of sarcopenia, that to obtain a comparison with other trials and a certain generalizability of data, it is possible to combine SarQoL with a generic tool and therefore, obtain a more accurate proxy of treatment efficacy.

Regarding relevance of SarQoL, qualitative interview results indicated that items and the seven dimensions currently covered by SarQoL are relevant.

Finally, the comprehensibility of SarQoL was appropriate, as patients expressed a clear understanding of the questionnaire which was confirmed by an adequate response to the corresponding question. Experts also agreed on the comprehensibility of the formulation of the questions, instructions and proposed answers. Two patients asked for clarification of the words used in the questionnaire, but as this was only reported by two patients for two different words, this very small proportion was not considered to reflect inappropriate wording of the question. In addition, the clear understating of SarQoL is supported by the large body of evidence from translation studies of the SarQoL questionnaire worldwide [4]. Indeed, seventeen validation studies reported a high level of comprehensibility of SarQoL items/domains/instructions/format following cognitive debriefing pre-test. A total of 219 sarcopenic individuals (i.e. 20 participants in the original French version, 25 in the Serbian version, 20 in the Brazilian version, 19 in the Kannada version, 16 in the Lithuanian version, 15 in the Greek version, 14 in the Dutch version and 10 in the Chinese, Polish, Spanish, Taiwanese, Turkish, English, Ukrainian, Cantonese, and Korean versions) confirmed their comprehensibility [21–36] of SarQoL in its current version.

Although the fact that the COSMIN methodology was strictly followed in this study, some limitations could be highlighted. First, our sample size of patients consisted only of Belgian individuals which may not be representative of all the patients as the SarQoL questionnaire is used worldwide. Secondly, the representativeness should be influenced by the qualitative nature of this study. In fact, the results obtained in patients and experts only reflect the views of the participants interviewed and may not be fully representative of the target population. However, the data saturation achieved through data collection and analysis tends to confirm that the study is representative of how the phenomenon of quality of life in sarcopenia is experienced in a Belgian population.

The robust content validity demonstrated by the SarQoL questionnaire is further reinforced by the understanding that a deficiency in content validity can affect various measurement properties of the PROM. Irrelevant items have the potential to reduce the internal consistency, structural validity, and interpretability of the PROM, while the absence of key concepts can compromise validity and responsiveness [5]. Remarkably, so far twenty-four validation studies of SarQoL (i.e. 19 translation/validation studies and 5 studies aiming at assessing specific psychometric properties) involving a total of 4807 older individuals all around the world, consistently yielded excellent results across all psychometric properties [4, 37]. This underscores and reinforces the robust evidence supporting the content validity of this study.

## Conclusion

In conclusion, the current form of SarQoL provides robust evidence of content validity in evaluating the health-related quality of life in people with sarcopenia. The relevance, the comprehensibility and comprehensiveness of the dimensions and items of the questionnaire have been assessed as adequate patients with sarcopenia and sarcopenia’s experts. Taking together, evidence from patients and experts, the current study confirms the content validity of SarQoL according to the COSMIN standard for validating the content validity of a PROM. Additional scales aiming to explore other concepts such as depression or patient empowerment can be used alongside the SarQoL questionnaire if deemed necessary and relevant for a particular study.

### Electronic supplementary material

Below is the link to the electronic supplementary material.

Supplementary Material 1

## Data Availability

No datasets were generated or analysed during the current study.
