# Supplementary material for: Content validity of SarQoL, a quality of life questionnaire specific to sarcopenia
Source: Aging Clin Exp Res. 2024 Apr 30;36(1):101. doi: 10.1007/s40520-024-02756-0 (PMC11074221; doi:10.1007/s40520-024-02756-0)
Supplement: Supplementary file 1 — Supplementary Material 1 [file 40520_2024_2756_MOESM1_ESM.docx]

Supplementary material

Table 1. Frequencies of item and dimensions elicited from patient and expert perspectives.

|  | Patient elicitation  (n, %) | Expert elicitation  (n, %) | Already covered in SarQoL + name of domain | Not covered but considered as part of a domain or subdomain | Not covered, considered as a not covered dimension |
| --- | --- | --- | --- | --- | --- |
| **Dimension 1: Physical and Mental Health** | **3 (17.6%)** | **8 (72.7%)** |  |  |  |
| Feeling frail |  | 2 (18.1%) | Yes, D1 Physical and Mental Health |  |  |
| Feeling weak |  | 2 (18.1%) | Yes, D1 Physical and Mental Health |  |  |
| Feeling tired |  | 3 (27.2%) | Yes, D1 Physical and Mental Health |  |  |
| **D2 Locomotion** | **4 (23.5%)** | **5 (45.4%)** |  |  |  |
| Limitation in the walking distance | 2 (11.8%) | 3 (27.2%) | Yes, D2 Locomotion |  |  |
| Limitation to go out walking |  | 2 (18.1%) | Yes, D2 Locomotion |  |  |
| Mobilisation skills |  | 3 (27.2%) |  | Yes, D2 Locomotion |  |
| **D3 Body Composition** | **1 (5.9%)** | **0 (0%)** |  |  |  |
| **D4 Functionality** | **3 (17.6%)** | **5 (45.4%)** |  |  |  |
| Falls |  | 4 (36.3%) | Yes, D4 functionality |  |  |
| **D5 Activities of daily living** | **9 (52.9%)** | **11 (100%)** |  |  |  |
| Carrying heavy objects |  | 2 (18.1%) | Yes, D5 Activities of daily living |  |  |
| Doing the housework | 2 (11.8%) | 4 (36.3%) | Yes, D5 Activities of daily living |  |  |
| Shopping/groceries |  | 7 (63.6%) | Yes, D5 Activities of daily living |  |  |
| Undertaking light physical activities |  | 5 (45.4%) | Yes, D5 Activities of daily living |  |  |
| Getting tired | 3 (17.6%) |  | Yes, D5 Activities of daily living |  |  |
| Need of assistance | 4 (23.5%) |  |  | Yes, D5 Activities of daily living |  |
| Hygiene cares |  | 4 (36.3%) |  | Yes, D5 Activities of daily living |  |
| Cooking |  | 2 (18.1%) |  | Yes, D5 Activities of daily living |  |
| **D6 Leisure activities** | **6 (35.3%)** | **7 (63.6%)** |  |  |  |
| Sharing time/go out with other people | 2 (11.8%) | 3 (27.2%) | Yes, D6 Leisure activities |  |  |
| Doing hobbies | 5 (29.4%) | 2 (18.1%) | Yes, D6 Leisure activities |  |  |
| Travelling | 3 (17.6%) |  | Yes, D6 Leisure activities |  |  |
| Social isolation |  | 4 (36.3%) |  | Yes, D6 Leisure activities |  |
| **D7 Fears** | 2 (11.8%) |  |  |  |  |
| Fear of falling |  | 3 (27.2%) | Yes, D7 Fears |  |  |
| Fear of the future | 3 (17.6%) |  |  | Yes, D7 Fears |  |
| **New items not covered by existing dimensions** |  |  |  |  |  |
| Depression | 0 (0%) | 3 (27.2%) |  |  | YES, “depression” |
| Adaptation and use of strategies | 8 (47.0%) |  |  |  | YES, “patient empowerment” |
| Acceptance of reduced state | 2 (11.7%) |  |  |  | YES, “patient empowerment” |
| Self-fulfillment | 5 (29.4%) |  |  |  | YES, “patient empowerment” |
| Increased dependency |  | 4 (36.3%) |  |  | YES, “patient empowerment” |

Table 2. Items relevance and comprehension from expert perspective

|  | Mean and range score of relevance (lowest-highest) | Mean and range score of comprehension(lowest-highest) |
| --- | --- | --- |
|  |  |  |
| Item 1: Reduction of strength in arms | 4.0 (4-4) | 3.7 (3-4) |
| Item 2: Reduction of strength in legs | 4.0 (4-4) | 3.7 (3-4) |
| Item 3: Reduction of muscle mass | 3.4 (2-4) | 3.7 (3-4) |
| Item 4: Reduction of energy | 3.7 (3-4) | 3.7 (3-4) |
| Item 5: Reduction of physical capabilities | 4.0 (4-4) | 3.7 (3-4) |
| Item 6: Reduction of general flexibility | 3.5 (2-4) | 3.7 (3-4) |
| Item 7: Pain in muscles | 3.5 (2-4) | 3.7 (3-4) |
| Item 8: Difficulty when undertaking light physical activities | 4.0 (4-4) | 3.8 (3-4) |
| Item 9: Get tired when undertaking light physical activities | 4.0 (4-4) | 3.8 (3-4) |
| Item 10: Experience pain when undertaking light physical activities | 3.7 (2-4) | 3.8 (3-4) |
| Item 11: Difficulty when undertaking moderate physical activities | 4.0 (4-4) | 3.8 (3-4) |
| Item 12: Get tired when undertaking moderate physical activities | 4.0 (4-4) | 3.8 (3-4) |
| Item 13: Experience pain when undertaking moderate physical activities | 3.7 (2-4) | 3.8 (3-4) |
| Item 14: Difficulty when undertaking intense physical activities | 4.0 (4-4) | 3.8 (3-4) |
| Item 15: Get tired when undertaking intense physical activities | 4.0 (4-4) | 3.8 (3-4) |
| Item 16: Experience pain when undertaking intense physical activities | 3.7 (2-4) | 3.8 (3-4) |
| Item 17: Feeling old | 3.7 (3-4) | 3.6 (1-4) |
| Item 18: Feeling weakness in muscles | 3.8 (3-4) | 3.6 (1-4) |
| Item 19: Feeling physically weak | 3.8 (3-4) | 3.8 (3-4) |
| Item 20: Limitation in walking time | 4.0 (4-4) | 3.8 (3-4) |
| Item 21: Limitation outings for walking | 4.0 (4-4) | 3.8 (3-4) |
| Item 22: Limitation of walking distance | 4.0 (4-4) | 3.8 (3-4) |
| Item 23: Limitation of walking speed | 3.9 (3-4) | 3.8 (3-4) |
| Item 24: Limitation of length of step | 3.6 (2-4) | 3.8 (3-4) |
| Item 25: Feeling tired when walking | 4.0 (4-4) | 4.0 (4-4) |
| Item 26: Need to sit down regularly to recover when walking | 4.0 (4-4) | 4.0 (4-4) |
| Item 27: Difficulty crossing roads quickly enough | 3.8 (2-4) | 4.0 (4-4) |
| Item 28: Difficulties with uneven surfaces | 3.8 (2-4) | 4.0 (4-4) |
| Item 29: Problems with balance | 3.8 (3-4) | 4.0 (4-4) |
| Item 30: Falls | 3.9 (3-4) | 3.7 (2-4) |
| Item 31: Modification of physical appearance | 3.6 (2-4) | 4.0 (4-4) |
| Item 32: Loss of muscle mass | 3.6 (3-4) | 3.9 (3-4) |
| Item 33: Feeling frail | 3.7 (2-4) | 3.6 (2-4) |
| Item 34: Climbing a flight of stairs | 4.0 (4-4) | 3.7 (2-4) |
| Item 35: Climbing several flights of stairs | 3.9 (3-4) | 3.7 (2-4) |
| Item 36: Going up one or several steps without holding on to the banister | 3.8 (3-4) | 3.7 (2-4) |
| Item 37: Squatting or kneeling | 3.9 (3-4) | 3.7 (2-4) |
| Item 38: Stooping or leaning down to pick up an object off the floor | 3.8 (2-4) | 3.7 (2-4) |
| Item 39: Getting up from the floor without holding on to anything | 4.0 (4-4) | 3.7 (2-4) |
| Item 40: Getting out of a low chair without armrests | 4.0 (4-4) | 3.7 (2-4) |
| Item 41: Moving from a sitting position to a standing position? | 3.8 (2-4) | 3.7 (2-4) |
| Item 42: Carrying heavy object | 4.0 (4-4) | 3.7 (2-4) |
| Item 43: Opening a bottle or a jar | 3.9 (3-4) | 3.7 (2-4) |
| Item 44: Using public transport | 3.5 (2-4) | 3.7 (2-4) |
| Item 45: Getting in or out of a car | 3.9 (3-4) | 3.7 (2-4) |
| Item 46: Shopping | 3.8 (3-4) | 3.7 (2-4) |
| Item 47: Doing the housework | 3.9 (3-4) | 3.7 (2-4) |
| Item 48: Limitation of movement | 3.9 (3-4) | 3.6 (1-4) |
| Item 49: Fear of pain | 3.9 (3-4) | 3.9 (3-4) |
| Item 50: Fear to not be able | 3.9 (3-4) | 3.9 (3-4) |
| Item 51: Fear of feeling tired | 3.9 (3-4) | 3.9 (3-4) |
| Item 52: Fear of falling | 3.9 (3-4) | 3.9 (3-4) |
| Item 53: Limitation of sex life | 3.8 (3-4) | 3.6 (1-4) |
| Item 54: Modification in physical activities/sports | 3.8 (3-4) | 4.0 (4-4) |
| Item 55: Modification in leisure activities | 3.9 (3-4) | 3.8 (2-4) |
